# Expectations of doctoral students in the field of medicine and health sciences towards a graduate school: an online cross-sectional survey in Germany

**DOI:** 10.3389/fmed.2024.1481796

**Published:** 2024-12-05

**Authors:** Beate Stock-Schröer, Silke Lange

**Affiliations:** ^1^Interprofessional Graduate School for Integrative Medicine and Health, Health Department, Witten/Herdecke University, Witten, Germany; ^2^Integrated Curriculum for Anthroposophic Medicine, Health Department, Witten/Herdecke University, Witten, Germany

**Keywords:** scientific education, dissertation, PhD, medical doctor, faculty development, complementary and integrative medicine and health

## Abstract

The doctoral thesis in medicine is a special case, as it is usually started during the course of study and the students have no experience of scientific work. This lack of scientific training is often criticized, and the quality of doctoral theses in medicine is considered low. In order to increase the quality of doctoral theses and the successful completion rate, a structured doctoral programme can provide the appropriate support. A cross-sectional survey of doctoral students in the field of medicine and health sciences was conducted to assess their expectations of a structured programme offered by a graduate school planned for doctoral studies in complementary and integrative medicine (CIM). Among other questions concerning experiences in their doctorate, the participants were asked to indicate their expectations in two free text answers and 24 pre-defined answers (5-point Likert scale). In addition, participants were asked how supervision should be organized in the curriculum. The majority of the participants expected individual personal support and advice in the graduate school, while financial support was not very important for them. In addition to the scientific training, networking and support among the participants of the graduate school was considered important. The non-medical participants were interested in personality development in science and networking with other doctoral students, whereas the medical doctoral students were more interested in scientific guidance. Doctoral students with a CIM topic expected a predicate and a better final grade by participating in such a graduate school. These results provide important information on how the curriculum in the graduate school should be designed. The next steps will be to evaluate the preliminary curriculum in order to develop a curriculum following the six-step approach of Kern.

## 1 Introduction

Worldwide 1.1% of the population on average in OECD countries have a doctorate, with the number of medical doctorates far outstripping those in other disciplines. From all doctorate holders Slovenia has the highest rate, followed by Sweden, with Germany in sixth place. The report from 2019 notice that doctoral graduates cannot only pursue an academic career, but are also in demand in industry and other sectors of the economy. Finding a successful and safe career in academia is difficult in all countries and leads many doctoral graduates to seek career opportunities outside of academic research (1).

In general, the doctorate is not part of the basic qualification for the professions, but is an academic degree added to the master’s/diploma degree. A large proportion of university research is carried out in the context of doctoral studies, which contribute significantly to the reputation of universities (2). Despite this great importance for the universities, the supervision and guidance of doctoral research is not part of a structured programme, but is the responsibility of the scientific standards of the individual research groups (3). Doctorates, or their supervision, are recorded as achievement of universities and their successful completion is considered as another indicator of academic qualification and value in the nationwide university ranking (2). In addition, doctorates are also used as an indicator for the calculation of performance-related funding and are considered, for example, as criteria for the evaluation of research funding programmes (4). Therefore, the successful completion of doctoral graduation is a central and mandatory duty of academic institutions. Faculty and administrators should prevent attrition of doctoral students by developing mechanisms for topic selection, emphasizing sequential planning, addressing motivation, providing regular faculty guidance, and encouraging peer support like dissertation groups (5–7).

Given the importance of higher education, many students still drop out during their studies for several reasons (8–10). Numbers of discontinued doctoral thesis are not easy to find and there is a high number of unreported cases as some doctoral students quit in an early stage (11). Reliable, generalizable information on success and drop-out rates for doctorates in Germany is still not available (11, 12). There are a number of factors contributing to doctoral attrition. As far as dropping out is concerned, doctoral students without funding have the highest drop-out rate, while students with selective research grants have the lowest (13). Lack of supervision, inadequate training of both students and supervisors, methodological problems and personal differences are important reasons for attrition (14–16). These challenges can be exacerbated by a lack of statistical support (14, 17) as well as the feeling of being exploited by the supervisor during the doctoral period (14) or not supported due to internal rivalries of research groups (18). Both the positive and negative aspects and the influence of supervisors need to be recognized by themselves (19) as the emotional wellbeing of doctoral students are connected to the supervisor (20). A high workload and psychological stress are common challenges with doctoral students reporting burnout (21), higher levels of depression, anxiety and stress (22). A meaningful research project, supportive relationship between supervisor and doctoral student, a sense of progress and limited suffering are crucial factors in whether students complete or drop out of their doctoral thesis (23–26).

Until a few years ago, medical students pursuing a doctorate in medicine lacked solid basic science training. Many scientific organizations have therefore called for quality-improving changes in recent years (27, 28). The scientific level of the doctoral theses accompanying the studies has been repeatedly criticized by the German Council of Science and Humanities, among others, and in a European comparison the German medical doctorate is not regarded as proof of independent research (28). Moreover, it is repeatedly criticized for its lack of quality not only in Germany (29, 30). Although very few medical graduates work in research after their studies in Germany, the doctorate rate for human medicine and health sciences graduates remains high with about 60, and 52% for veterinary medicine graduates (3).

Research projects and critical thinking skills are crucial for medical students and physicians to improve their professional competence and contribute to the advancement of medical knowledge (31). Moreover the indirect and implicit benefit of a doctorate can be seen in the fact that doctoral graduates have developed personally as a result of their doctoral thesis and feel more academically competent for their future career as physicians (32).

The doctorate in medicine (Dr. med.) is a special case in Germany compared to other doctorates, as it can usually be started during the actual medical studies and no official degree is required, e.g., in the form of a Master’s thesis for admission to a doctorate. However, the doctoral thesis may only be submitted after the license to practise medicine has been granted. Most of medical doctoral students (85%) are doing their doctorate because it is common for physicians and 75% believe that a doctorate improves their job opportunities (32). In the field of health sciences academic careers are more in the focus of the doctoral candidates but still there is a lack of career options in universities (33). In Germany, different titles are possible at the end of a doctorate. In the field of medicine and health sciences, which is an interdisciplinary field, students without a specific medical degree can also study for a doctorate at medical faculties, but will receive doctoral degrees such as Dr. rer. medic or Dr. hum. biol. e.g., (34).

Complementary and integrative therapies continue to be very popular among the population in Europe (35, 36) with no predictive factors for why patients visit a therapist specializing in CIM (37). Latest numbers for Germany indicate that 70% of the respondents reported that they had used CIM at some time during their lives (38). Although there is increasing evidence of positive effects on health in several areas for example in the treatment of nausea and vomiting during pregnancy (39) or in the treatment of patients in oncology (40), these authors conclude that quality and number of studies included in the reviews were poor and more studies are needed. The lack of studies in the field of CIM in Germany is related to the very small number of faculties with the expertise and willingness to conduct research on CIM topics. In addition to facilities, finances are a limiting factor too (41). As CIM research projects often are conducted within the scope of doctoral theses there is a great need for a thorough training and guidance for doctoral students in science. One successful opportunity was demonstrated by the cooperation between two faculties that taught CIM students in scientific training programmes as part of a mentoring programme (42).

Another option to improve scientific training is to implement a structured doctoral programme for doctoral students with a CIM topic. The figures for the number of doctoral students in structured doctoral programmes at German universities still vary widely between 19 and 42% depending on the data source and study used. Referring to the numbers of the latest report in Germany, around 40% of doctoral students in medicine and health sciences took part in structured doctoral programmes in 2019 (43). In Germany, structured doctoral programmes are usually offered in Graduate Schools, which tend to be thematically focused. A structured doctorate is characterized by official enrollment in a doctoral programme, regular supervision by several university lecturers and a compulsory range of courses (44). The number of courses and how many have to be attended, the thematic focus, whether colloquia or scientific lessons and the frequency can vary (45).

Several faculties in Germany have established medical research schools as structured doctoral programmes. So far, there is no such programme for CIM. Since CIM is constantly under great scrutinity due to the lack of high quality studies and evidence, a structured doctoral programme is needed to improve the quality of dissertations and promote young researchers. At the Witten/Herdecke University, an inter-professional and inter-faculty graduate school was to be established for all doctoral students interested in complementary and integrative medicine (CIM). In advance, this survey was designed to provide detailed information on the needs of potential participants in order to start developing a curriculum.

### 1.1 Aim of this study

This study was conducted to analyze

1. what benefits doctoral students expect from a structured doctoral programme in a graduate school,
2. what suggestions they have for the content and timing of the curriculum,
3. whether doctoral students with a topic in the field of CIM differ from those with a Non-CIM topic regarding the expectations,
4. whether there are differences between medical and non-medical doctoral students regarding the expectations.

## 2 Materials and methods

A cross sectional online survey was conducted among doctoral students via LimeSurvey (LimeSurvey GmbH, n.d.) from October 1, 2020 to December 31, 2020. Students from the health sector - mainly medical students - in Germany were invited to participate. Inclusion criteria for the study were that participants were currently pursuing or had completed a doctoral degree. The questionnaire explicitly addressed doctoral students with a topic in CIM. Recruiting was carried out through email distribution lists of the medical departments and faculties in Germany, German medical education association GMA and the *Forum universitärer Arbeitsgruppen* - an association of working groups at medical faculties specialized on C*omplementary and Integrative Medicine* (CIM). Mail recipients were asked to forward the request for participation in the survey to their doctoral student mailing lists. As the latter group of recipients is known for research in the field of CIM, the target group of CIM doctoral students were reached via this mailing list to answer the questionnaire.

The questionnaire was self developed through a systematic process including literature review, expert knowledge gained from existing medical research schools, pre-testing (think-aloud-method) with medical students and revision after the pretest. The pre-test was used to check the comprehensibility of the questions, which were then linguistically adapted. In order to identify the needs of doctoral students for good supervision in the graduate school, questions have been compiled on skills that are considered fundamental to scientific work. These include skills such as academic writing, literature research and administration, and the use of software programmes. On the other hand, questions were formulated that are cited in the literature as reasons for dropping out of a doctoral programme and that may be helpful in the context of a graduate school: Finances, support both within the team and from a statistician, career opportunities, e.g., the questionnaire consisted of 35 questions divided into the following main topics: 1. Experiences of doctoral students working on their theses, 2. Identifying and describing difficulties and factors for doctoral success, 3. Expectations of structured supervision in a CIM-focused graduate school.

For this piece of work, results of the third field in the questionnaire, consisting of six questions, were analyzed (Supplementary Material 1). A pre-defined list of 24 general expectations and scientific competencies that can be achieved in graduate school was given, supplemented by spaces for free text responses. The competencies listed are in the following areas: research methodology, software training, epistemological and health theory approaches, the various therapy approaches, literature research and managing, personal abilities like communication skills and conflict management as well as promotion of personality development in science. In addition, the following expectations could be stated: Finding a professional perspective and/or career in science, individual guidance for scientific work, personal support by a statistician, networking and interdisciplinary cooperation with other doctoral students, mutual support and motivation in the doctoral team to write and keep going, support in case of arising questions or problems, quality improvement of the doctoral thesis and a better final degree grade, covering costs (all or travel expenses or material costs) related to the curriculum of the graduate school, a scholarship programme of its own as well as receiving a predicate due to participation.

Participants could rate these predefined skills in the list on a Likert scale from 1 (strongly disagree) to 5 (strongly agree). Descriptive statistics were carried out (SPSS 27.0). Chi-square tests were calculated to compare two independent groups with regard to the distribution of a categorial variable. The resulting *p*-values were interpreted as strictly descriptive.

## 3 Results

### 3.1 Study collective

In all, 246 participants saved their answers in the online tool. 162 data sets were completed and used for the evaluation of the study. Most participants were female (64.8%, *n* = 105), about one-third were male (33.9%, *n* = 55), two (1.2%) did not provide gender information. On average, participants were 36 years old (22–66 years, median 32). Stated disciplines were assigned to six fields of study. Most participants, 54.3%, studied medicine (*n* = 88) including dentistry (*n* = 5) and veterinary medicine (*n* = 3), 18.5% (*n* = 30) studied health sciences, and 12.3% (*n* = 20) natural sciences. Only 6.2% of the participants studied nursing sciences (*n* = 10), 4.3% psychology (*n* = 7) and 4.3% CIM (*n* = 7). Most participants - 62.3% (*n* = 101) - reported that they were studying for a doctorate at the time of the survey, 35.8% (*n* = 58) had already completed their thesis, two intended to do a doctorate and one canceled. 162 participants answered the question about their professional situation. 29.6% participants (*n* = 48) stated that they were employed full-time during their doctorate. 21.0% (*n* = 34) reported being employed by the institution they are doing their doctorate. Another 21.0% (*n* = 34) participants were following their doctorate parallel with their studies. 16.0% (*n* = 26) were employed part-time during their doctorate, 10.5% (*n* = 17) were released from work to do their doctorate. 131 participants answered how much time they spend on their doctoral studies. The average was 20.3 h per week (1–60 h, median 16). 37.6% (*n* = 61) participants were working on a doctorate with a topic from the CIM area, while 62.3% (*n* = 101) were working on a topic that cannot be assigned to CIM (Non-CIM). Topics from CIM were assigned to the following areas (Table 1). Multiple entries were possible.

**TABLE 1:** Frequencies of CIM areas in doctorates for the total sample and the subgroup of medical students (multiple entries possible).

Topic of the doctorate belongs to the following area:	Total sample	Medical doctoral students
Naturopathy	18	9
Anthroposophic medicine	17	14
Phytotherapy	10	7
Relaxation techniques	8	3
TCM	5	4
Yoga	5	3

### 3.2 Expectations toward a graduate school (total sample)

**FIGURE 1:**
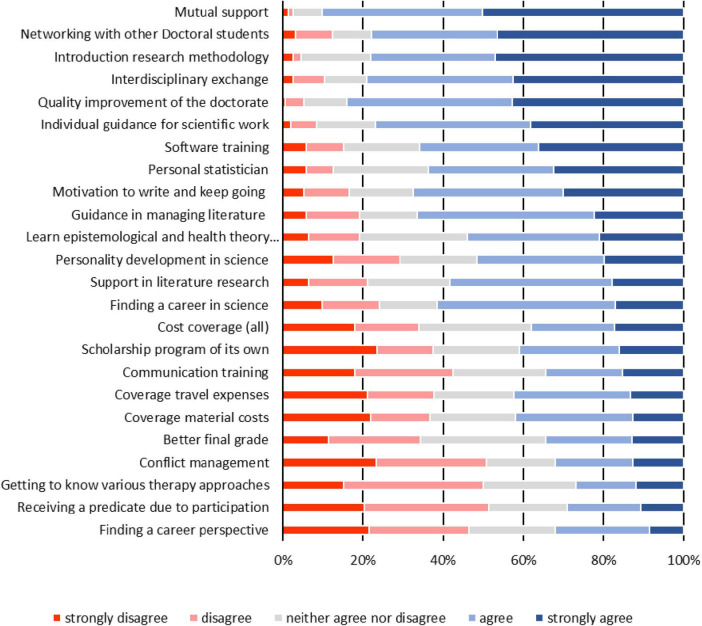
Expectations of a graduate school (5-point-Likert scales “strongly disagree” to “strongly agree”) - total sample.

In all, between 148 and 153 participants answered the single questions of the predefined list. The percentages listed below refer to the combined answers “strongly agree” and “agree.” The majority (90.2%, *n* = 138) expected a support among the participants of the graduate school, when questions and problems are arising, 77.8% (*n* = 119) were hoping for networking with other doctoral students and 79.1% (*n* = 121) for an interdisciplinary exchange in the group. Beside these demands for a support within the group of the doctoral students, 78.1% of the participants (*n* = 118) wished to have an introduction into research methodology. 77.0% (*n* = 117) hoped to receive support in the graduate school in the form of individual guidance for scientific work and a statistician as a personal contact (63.6%, *n* = 96). The graduate school should also provide competencies like software trainings (65.8%, *n* = 100) and support in finding (58.3%, *n* = 88) and managing literature (66.4%, *n* = 101). Only 32.0% (*n* = 49) expected to find a professional perspective being attendee in the graduate school whereas 61.4% (*n* = 94) expect finding a career in science. To receive a predicate as being an attendee was only important for 29.0% of the participants (*n* = 43) and for 38.0% getting a coverage of the costs (*n* = 57). Further results shown in Figure 1.

In order to get an idea of how the curriculum of the graduate school should be structured from the participants’ point of view, the participants were asked to indicate, at which location, how often and in what form they would participate in education events/colloquia. A third of the participants (35.0%, *n* = 55) expected the curriculum of the graduate school taking place at different university locations. The question about the frequency and type of events showed that the participants would prefer on average four face-to-face conferences (1–54, Median 4), six video conferences (0–52, Median 6) and two face-to-face events at other universities per year (0–27, Median 2). The majority (79.6%, *n* = 125) expected individual personal support and consultation in the graduate school. 28.0% (*n* = 44) of participants expected the curriculum and supervision to be primarily group-based. For 37.5% (*n* = 59), it was important to include units that serve personality development, such as modules on stress management or biography work.

### 3.3 Different expectations of medical doctoral students with CIM and non-CIM topics

**FIGURE 2:**
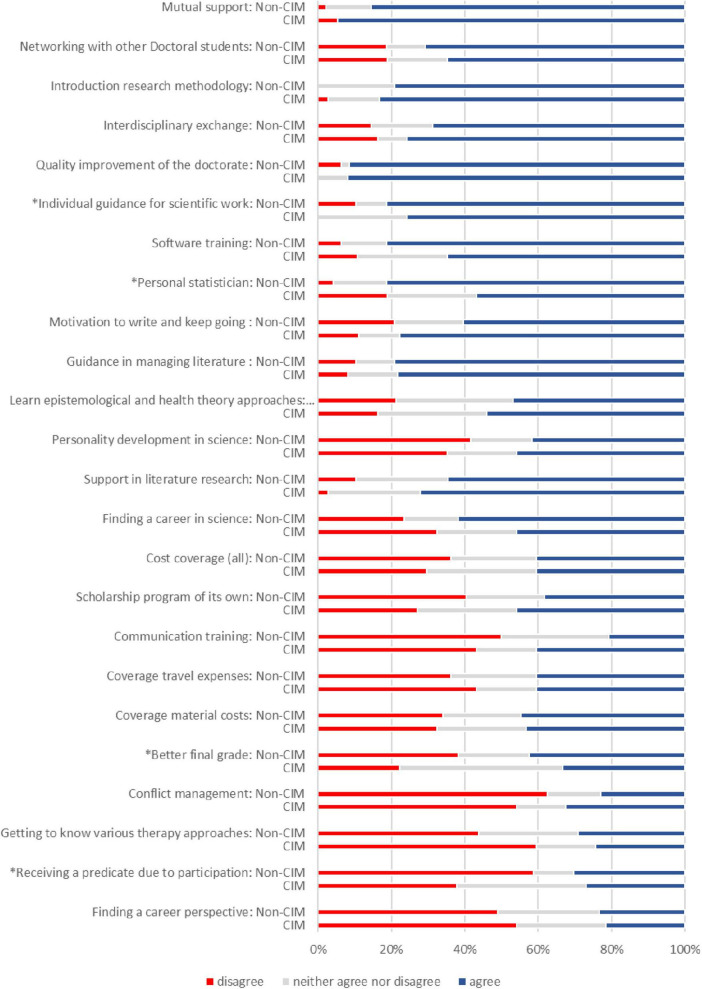
Expectations of a graduate school (5-point-Likert scales “strongly disagree” to “strongly agree”) - comparison of medical doctoral students with CIM and Non-CIM topic. For this figure the categories “strongly disagree” and “disagree” were combined into “disagree,” “strongly agree” and “agree” were combined to “agree.” *Indicates group differences (*p* ≤ 0.05).

We investigated whether the choice of topic made a difference in the expectations within the group of medical doctoral students (*n* = 88). 44.3% (*n* = 39) of them worked on a topic assigned to CIM and 55.6% (*n* = 49) not related to a CIM topic. As group size was small we condensed the 5 point Likert scale to three categories (agree, partly and not agree) for the comparison. 81.3% doctoral students with a Non-CIM topic (*n* = 39) asked for more individual guidance for scientific work than CIMs (75.7%, *n* = 28) (*p* = 0.024). An individual guidance from a statistician was also more important for 81.3% Non-CIMs (*n* = 39) and only for 56.8% of the CIMs (n = 21) (*p* = 0.028). A better final grade and receiving a predicate being member in the graduate school was less important for the Non-CIMs [not agree: 38.3% (*n* = 18) and 58.7% (*n* = 27)] than for the CIMs [not agree: 22.2% (*n* = 8) and 37.8% (*n* = 14)] (*p* = 0.04, *p* = 0.024) (Figure 2 and Table 2).

**TABLE 2:** Expectations of a graduate school (frequencies and test statistics) - medical doctoral students with CIM and non-CIM topic.

Expectation	Group	Disagree *n* (%)	Neither agree nor disagree *n* (%)	Agree *n* (%)	χ ^2^(2)	*p*
Finding a career perspective	CIM	20 (54.1)	9 (24.3)	8 (21.6)	0.223	0.895
	Non-CIM	23 (48.9)	13 (27.7)	11 (23.4)		
Finding a career in science	CIM	12 (32.4)	8 (21.6)	17 (45.9)	2.080	0.354
	Non-CIM	11 (23.4)	7 (14.9)	29 (61.7)		
Individual guidance for scientific work	CIM	0 (0)	9 (24.3)	28 (75.7)	7.430	0.024
	Non-CIM	5 (10.4)	4 (8.3)	39 (81.3)		
Support in literature research	CIM	1 (2.8)	9 (25)	26 (72.2)	1.857	0.395
	Non-CIM	5 (10.4)	12 (25)	31 (64.6)		
Guidance in managing literature	CIM	3 (8.1)	5 (13.5)	29 (78.4)	0.290	0.865
	Non-CIM	5 (10.4)	5 (10.4)	38 (79.2)		
Introduction research methodology	CIM	1 (2.8)	5 (13.9)	30 (83.3)	1.933	0.380
	Non-CIM	0 (0)	10 (20.8)	38 (79.2)		
Software training	CIM	4 (10.8)	9 (24.3)	24 (64.9)	2.940	0.230
	Non-CIM	3 (6.3)	6 (12.5)	39 (81.3)		
Communication training	CIM	16 (43.2)	6 (16.2)	15 (40.5)	4.451	0.108
	Non-CIM	24 (50)	14 (29.2)	10 (20.8)		
Conflict management	CIM	20 (54.1)	5 (13.5)	12 (32.4)	0.970	0.616
	Non-CIM	30 (62.5)	7 (14.6)	11 (22.9)		
Personality development in science	CIM	13 (35.1)	7 (18.9)	17 (45.9)	0.378	0.828
	Non-CIM	20 (41.7)	8 (16.7)	20 (41.7)		
Getting to know various therapy approaches	CIM	22 (59.5)	6 (16.2)	9 (24.3)	2.304	0.310
	Non-CIM	21 (43.8)	13 (27.1)	14 (29.2)		
Networking with other Doctoral students	CIM	7 (18.9)	6 (16.2)	24 (64.9)	0.652	0.722
	Non-CIM	9 (18.8)	5 (10.4)	34 (70.8)		
Mutual support	CIM	2 (5.4)	0 (0)	35 (94.6)	5.475	0.065
	Non-CIM	1 (2.1)	6 (12.5)	41 (85.4)		
Interdisciplinary exchange	CIM	6 (16.2)	3 (8.1)	28 (75.7)	1.359	0.507
	Non-CIM	7 (14.6)	8 (16.7)	33 (68.8)		
Learn epistemological and health theory approaches	CIM	6 (16.2)	11 (29.7)	20 (54.1)	0.528	0.768
	Non-CIM	10 (21.3)	15 (31.9)	22 (46.8)		
Motivation to write and keep going	CIM	4 (11.1)	4 (11.1)	28 (77.8)	2.856	0.240
	Non-CIM	10 (20.8)	9 (18.8)	29 (60.4)		
Personal statistician	CIM	7 (18.9)	9 (24.3)	21 (56.8)	7.124	0.028
	Non-CIM	2 (4.2)	7 (14.6)	39 (81.3)		
Quality improvement of the doctorate	CIM	0 (0)	3 (8.1)	34 (91.9)	3.917	0.141
	Non-CIM	3 (6.4)	1 (2.1)	43 (91.5)		
Better final grade	CIM	8 (22.2)	16 (44.4)	12 (33.3)	6.462	0.040
	Non-CIM	18 (38.3)	9 (19.1)	20 (42.6)		
Receiving a predicate due to participation	CIM	14 (37.8)	13 (35.1)	10 (27)	7.456	0.024
	Non-CIM	27 (58.7)	5 (10.9)	14 (30.4)		
Cost coverage (all)	CIM	11 (29.7)	11 (29.7)	15 (40.5)	0.574	0.751
	Non-CIM	17 (36.2)	11 (23.4)	19 (40.4)		
Coverage material costs	CIM	12 (32.4)	9 (24.3)	16 (43.2)	0.111	0.946
	Non-CIM	16 (34)	10 (21.3)	21 (44.7)		
Coverage travel expenses	CIM	16 (43.2)	6 (16.2)	15 (40.5)	0.792	0.673
	Non-CIM	17 (36.2)	11 (23.4)	19 (40.4)		
Scholarship program of its own	CIM	10 (27)	10 (27)	17 (45.9)	1.655	0.437
	Non-CIM	19 (40.4)	10 (21.3)	18 (38.3)		

The categories “strongly disagree” and “disagree” were combined into “disagree,” “strongly agree” and “agree” were combined to “agree.” χ^2^(2) = test statistic of Chisquare test, p = *p*-value of Chisquare test.

### 3.4 Different expectations of medical and non-medical doctoral students

As medical students may request different support than students from other disciplines, participants were subdivided in two groups: The following compares 88 medical doctoral students (*Medicals*) and 74 non-medical (*Non-Medicals*).

The main differences between the two groups are that Medicals expect more support in terms of methodology and software training: 67.9% of Medicals (*n* = 57) wanted help in researching for literature unlike 46.3% of Non-Medicals (*n* = 31) (*p* < 0.001). 78.8% Medicals (*n* = 67) need support for managing the literature and only 50.7% of Non-Medicals (*n* = 34) (*p* < 0.001). Software training is needed by 74.1% Medicals (*n* = 63) and 55.2% Non-Medicals (*n* = 37) (*p* = 0.028). 91.7% of the Medical Doctoral students (*n* = 77) expect by training and supervision in the graduate school a quality improvement of the doctoral thesis whereas 74.2% of the Non-Medicals do so (*n* = 49) (*p* = 0.027).

**FIGURE 3:**
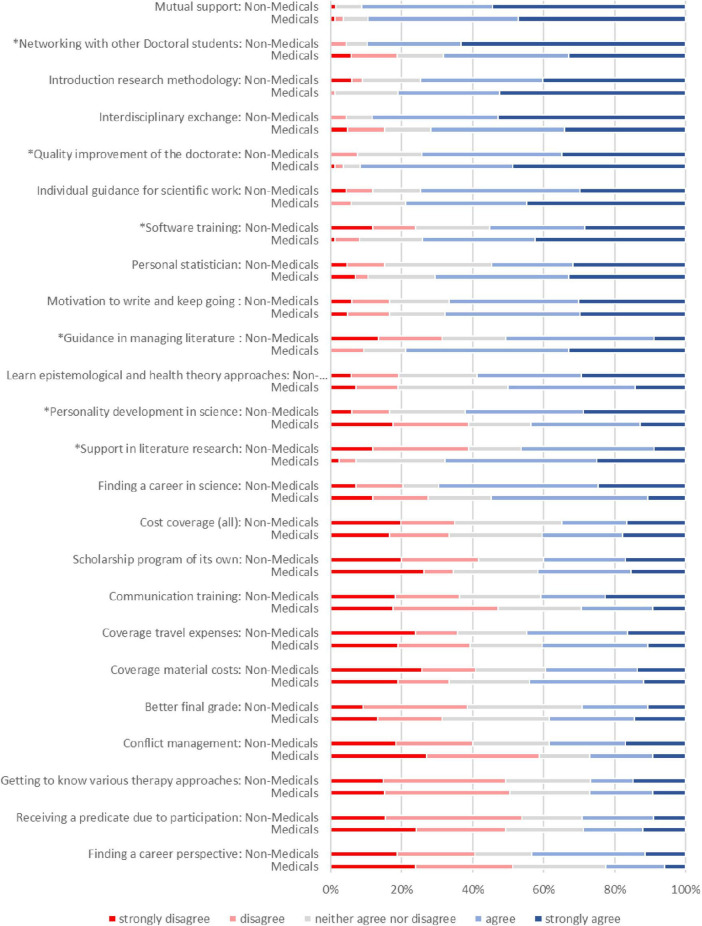
Expectations of a graduate school (5-point-Likert scales “strongly disagree” to “strongly agree”) - comparison of medical and non-medical doctoral students. *Indicates group differences (*p* ≤ 0.05).

Non-Medicals have a greater need for networking in the group and personality development in science through graduate school: 38.8% Medicals (*n* = 33) did not see a great need for the support of the development of personality in science in contrast to 62.1% of the Non-Medicals (*n* = 41) who consider it very important (*p* = 0.021). 89.7% Non-Medicals (*n* = 61) see networking with other doctoral students as important, while only 68.2% Medicals (*n* = 58) indicated this (*p* = 0.002). 88.2% of the Non-Medicals (*n* = 60) also rate interdisciplinary cooperation very important whereas less Medicals do so (71.8%, *n* = 61) (*p* = 0.052) (Figure 3 and Table 3).

**TABLE 3:** Expectations of a graduate school (frequencies and test statistics) - medical and non-medical doctoral students.

Expectation	Group	Strongly disagree *n* (%)	Disagree *n* (%)	Neither agree nor disagree *n* (%)	Agree *n* (%)	Strongly agree *n* (%)	χ ^2^ (4)	*p*
Finding a career perspective	Medicals	20 (23.8)	23 (27.4)	22 (26.2)	14 (16.7)	5 (6)	7.911	0.095
	Non-medicals	13 (18.8)	15 (21.7)	11 (15.9)	22 (31.9)	8 (11.6)		
Finding a career in science	Medicals	10 (11.9)	13 (15.5)	15 (17.9)	37 (44)	9 (10.7)	6.890	0.142
	Non-medicals	5 (7.2)	9 (13)	7 (10.1)	31 (44.9)	17 (24.6)		
Individual guidance for scientific work	Medicals	0 (0)	5 (5.9)	13 (15.3)	29 (34.1)	38 (44.7)	7.301	0.121
	Non-medicals	3 (4.5)	5 (7.5)	9 (13.4)	30 (44.8)	20 (29.9)		
Support in literature research	Medicals	2 (2.4)	4 (4.8)	21 (25)	36 (42.9)	21 (25)	25.134	<0.001
	Non-medicals	8 (11.9)	18 (26.9)	10 (14.9)	25 (37.3)	6 (9)		
Guidance in managing literature	Medicals	0 (0)	8 (9.4)	10 (11.8)	39 (45.9)	28 (32.9)	24.231	<0.001
	Non-medicals	9 (13.4)	12 (17.9)	12 (17.9)	28 (41.8)	6 (9)		
Introduction research methodology	Medicals	0 (0)	1 (1.2)	15 (17.9)	24 (28.6)	44 (52.4)	7.218	0.125
	Non-medicals	4 (6)	2 (3)	11 (16.4)	23 (34.3)	27 (40.3)		
Software training	Medicals	1 (1.2)	6 (7.1)	15 (17.6)	27 (31.8)	36 (42.4)	10.840	0.028
	Non-medicals	8 (11.9)	8 (11.9)	14 (20.9)	18 (26.9)	19 (28.4)		
Communication training	Medicals	15 (17.6)	25 (29.4)	20 (23.5)	17 (20)	8 (9.4)	6.317	0.177
	Non-medicals	12 (18.2)	12 (18.2)	15 (22.7)	12 (18.2)	15 (22.7)		
Conflict management	Medicals	23 (27.1)	27 (31.8)	12 (14.1)	15 (17.6)	8 (9.4)	5.675	0.225
	Non-medicals	12 (18.5)	14 (21.5)	14 (21.5)	14 (21.5)	11 (16.9)		
Personality development in science	Medicals	15 (17.6)	18 (21.2)	15 (17.6)	26 (30.6)	11 (12.9)	11.501	0.021
	Non-medicals	4 (6.1)	7 (10.6)	14 (21.2)	22 (33.3)	19 (28.8)		
Getting to know various therapy approaches	Medicals	13 (15.3)	30 (35.3)	19 (22.4)	15 (17.6)	8 (9.4)	1.820	0.769
	Non-medicals	10 (14.9)	23 (34.3)	16 (23.9)	8 (11.9)	10 (14.9)		
Networking with other doctoral students	Medicals	5 (5.9)	11 (12.9)	11 (12.9)	30 (35.3)	28 (32.9)	17.332	0.002
	Non-medicals	0 (0)	3 (4.4)	4 (5.9)	18 (26.5)	43 (63.2)		
Mutual support	Medicals	1 (1.2)	2 (2.4)	6 (7.1)	36 (42.4)	40 (47.1)	2.331	0.675
	Non-medicals	1 (1.5)	0 (0)	5 (7.4)	25 (36.8)	37 (54.4)		
Interdisciplinary exchange	Medicals	4 (4.7)	9 (10.6)	11 (12.9)	32 (37.6)	29 (34.1)	9.374	0.052
	Non-medicals	0 (0)	3 (4.4)	5 (7.4)	24 (35.3)	36 (52.9)		
Learn epistemological and health theory approaches	Medicals	6 (7.1)	10 (11.9)	26 (31)	30 (35.7)	12 (14.3)	5.784	0.216
	Non-medicals	4 (5.9)	9 (13.2)	15 (22.1)	20 (29.4)	20 (29.4)		
Motivation to write and keep going	Medicals	4 (4.8)	10 (11.9)	13 (15.5)	32 (38.1)	25 (29.8)	0.238	0.993
	Non-medicals	4 (6.1)	7 (10.6)	11 (16.7)	24 (36.4)	20 (30.3)		
Personal statistician	Medicals	6 (7.1)	3 (3.5)	16 (18.8)	32 (37.6)	28 (32.9)	7.928	0.094
	Non-medicals	3 (4.5)	7 (10.6)	20 (30.3)	15 (22.7)	21 (31.8)		
Quality improvement of the doctorate	Medicals	1 (1.2)	2 (2.4)	4 (4.8)	36 (42.9)	41 (48.8)	10.959	0.027
	Non-medicals	0 (0)	5 (7.6)	12 (18.2)	26 (39.4)	23 (34.8)		
Better final grade	Medicals	11 (13.3)	15 (18.1)	25 (30.1)	20 (24.1)	12 (14.5)	3.467	0.483
	Non-medicals	6 (9.2)	19 (29.2)	21 (32.3)	12 (18.5)	7 (10.8)		
Receiving a predicate due to participation	Medicals	20 (24.1)	21 (25.3)	18 (21.7)	14 (16.9)	10 (12)	4.282	0.369
	Non-medicals	10 (15.4)	25 (38.5)	11 (16.9)	13 (20)	6 (9.2)		
Cost coverage (all)	Medicals	14 (16.7)	14 (16.7)	22 (26.2)	19 (22.6)	15 (17.9)	0.847	0.932
	Non-medicals	13 (19.7)	10 (15.2)	20 (30.3)	12 (18.2)	11 (16.7)		
Coverage material costs	Medicals	16 (19)	12 (14.3)	19 (22.6)	27 (32.1)	10 (11.9)	1.524	0.822
	Non-medicals	17 (25.8)	10 (15.2)	13 (19.7)	17 (25.8)	9 (13.6)		
Coverage travel expenses	Medicals	16 (19)	17 (20.2)	17 (20.2)	25 (29.8)	9 (10.7)	2.915	0.572
	Non-medicals	16 (23.9)	8 (11.9)	13 (19.4)	19 (28.4)	11 (16.4)		
Scholarship program of its own	Medicals	22 (26.2)	7 (8.3)	20 (23.8)	22 (26.2)	13 (15.5)	5.810	0.214
	Non-medicals	13 (20)	14 (21.5)	12 (18.5)	15 (23.1)	11 (16.9)		

χ^2^(4) = test statistic of Chisquare test, p = *p*-value of Chisquare test.

### 3.5 Further comments in the free text sections

In addition, the participants had the opportunity to document in free text what else they expected from the graduate school. They expressed the wish for an independent contact person outside their own research group, who could also mediate between doctoral students and supervisors. The opportunity to think outside the box was mentioned several times, as well as the desire for peer learning, the provision of contacts to other research institutions, support in applying for research funding and ethics grants, and an alumni programme. Comments are listed in (Supplementary Table 2).

## 4 Discussion

In a cross-sectional survey of doctoral students in Germany, participants were asked to comment on structured doctoral programmes and to formulate their recommendations for a curriculum in a graduate school with a focus on CIM. The majority of the study participants expected a mutual support in the graduate school for questions and problems and more than two-thirds hoped for networking with other doctoral students and an interdisciplinary exchange within the group. The personal exchange and regular meetings of the members in a cross-faculty framework were desired. Regular face-to-face meetings, in addition to video calls, were considered important for the timing and structure of the curriculum. While the medical students attached more importance to courses with specific content on scientific skills, the non-medical students stated that they preferred the exchange with others. Doctoral students with a CIM topic hoped for a better final grade and a predicate for attending a graduate school, while the non-CIM doctoral students hoped for more individual guidance for scientific work and a perspective for a research career.

### 4.1 Strengths and limitations

The response rate (65.8%) was quite high due to a convenient distribution point, although the questionnaire appeared to be too long as many participants did not complete all parts of the whole questionnaire. The normal online survey response rate is 44.1% (46). The method of an online survey implies the problem of uncontrolled distribution and that people participate who are not part of the focus group in the first place (47). Initially, we intended to address only medical students or other health professions working on a topic in CIM for their doctorate. It turned out that other professions and doctoral students without a topic in CIM participated as well. This allowed us to compare subgroups in the second place.

The survey was scheduled for a short period of 3 months from October to December. A longer period might have resulted in a higher response rate and brought in further aspects from other participants. The sample is therefore not representative, but is intended to give an initial impression of what students might expect from a graduate school.

There is already a great deal of research on doctoral training in programmes and their various orientations: from interdisciplinarity to international courses (34). However, to date there have been no surveys on the wishes and expectations of doctoral students themselves with regard to structured doctoral supervision.

### 4.2 Improvement of quality in a structured doctoral programme

The quality of doctoral programmes is being discussed in many countries and efforts have already been made to improve doctoral training in such a programme in accordance with the respective doctoral regulations of the individual countries (34). In this study, more than 80% of the participants expect an improvement in the quality of the doctorate supervised in a graduate school. This is a high motivation for the participants to join a structured programme or an inter-faculty graduate school. In order to meet this expectation and to achieve the goal, the curriculum has to meet the needs.

To ensure the scientific training of students, there is no template for the curriculum, but mandatory things for a successful training are an adequate supervision, a sufficient time schedule and enough autonomy (48). These statements are in line with our results. In addition to course units that teach, mainly the medical students, the basic skills of scientific work, there must also be room for mutual exchange and individual support. This needs to be considered when designing the first curriculum as well as meeting the different needs of an interprofessional group. The non-medical doctoral students in this study emphasized the importance of being encouraged in graduate school to develop their personalities in order to find their career in academia.

### 4.3 Forms of support depending on topic (CIM or non-CIM) and disciplines

In this study, participants working on a non-CIM topic in particular wanted individual support from a graduate school by a statistician to better understand their own data. This is in line with the results of Can et al., according to which the majority of students lack statistical support (15). When considering the methods used in the doctoral projects with a CIM topic in this study, mainly literature searches and qualitative methods were reported, only a few studies were clinical or quantitative and required appropriate statistical analysis. This could be the reason why this group does not require intensive statistical support. Clinical studies, especially in the field of CIM, are difficult to carry out as part of a medical thesis, as they require not only an experienced research team, but also the appropriate inpatient or outpatient involvement and financial support. In Germany, there are only a few centers with large hospitals (Berlin, Tübingen, Essen, Witten e.g.,) where students can work in such a clinical environment with thematically experienced professional support. For those doctoral students who worked on a topic in CIM, a better final grade and the award of the predicate “member of the graduate school” were more important than the supervision itself.

Whereas the non-medical participants hope, that the graduate school will facilitate good interdisciplinary exchange, medical doctoral students hope for a good guidance in preparing their theses. This is not surprising, as the non-medical students have usually already written two theses (Bachelor’s and Master’s) in preparation for their doctoral thesis and have therefore already gained experience in scientific work.

### 4.4 Finances

Financial issues are one of the most challenging factors for doctoral students and are responsible for high dropout rates during doctoral studies (13, 23). Research assistants could benefit most from integration into such a structured doctoral programme, as their doctoral topic is often integrated into their work and at least partly paid for. External attendees whose job is not connected to the project have time problems in particular and feel more burdened by participating in such a programme (44). In this study, less than 20% participants wanted financial support in form of a scholarship. A cost coverage of expenses is expected by about 40% of the participants in this study either medical or non-medical.

### 4.5 Structured doctoral programme in a graduate school

Participating in a structured doctoral programme provides an improvement of quality, increasing of the completion rate (49), and facilitate academic career paths within and outside universities (3).

As a result of this study, running the programme in a graduate school offers the opportunity to build a network by bringing together young researchers at different levels of knowledge. Collaboration within the graduate school and networking with the scientific community are not the only important aspects. Attendees of the graduate school expect to develop skills that go beyond pure scientific training, such as conflict management or personal development. The opportunity for interdisciplinary exchange and the idea of networking in a graduate school was more important for the non-medical participants in this study than for the medical students. Medical doctoral students focused on the need to improve their scientific work as defined in the course content. This is in accordance to earlier investigations (50, 51) and needs be incorporated into the curriculum.

With regard to the interprofessional orientation of the curriculum, the expectations and needs of both non-medical and medical professionals need to be taken into account when designing the curriculum as well as the perspective of the supervisors. The supervisors’ assessments differ considerably from those of the doctoral students. While doctoral students more frequently cited difficulties with the research methodology or the dissertation topic in general as reasons for dropping out of the project, the supervisors suspected that the real reasons for dropping out were difficulties with time management and also reported personal differences with the students (15). While this piece of work focuses on the needs of doctoral students, in the next step, the attitudes and wishes of the supervisors are surveyed and analyzed. The input from supervisors will also be used in the development of the Graduate School, as will the regular evaluation of the Graduate School programme. As the process continues, these surveys and analyses will feed into the development of the curriculum. This development will follow Kern’s six-step approach (52).

## 5 Conclusion

The implementation of structured doctoral programmes in universities is a promising factor for improving the quality and completion rate of doctoral students worldwide. This study focused on the requirements for a curriculum, planned to establish a graduate school with expertise in CIM topics and in an interprofessional framework at Witten/Herdecke University. The expectations formulated by the participants themselves provide a good indication of the content and organizational structure of this graduate school. Although the topic of supervised doctoral students should belong to CIM, the general needs of students do not differ much from other topics. A good graduate school curriculum that offers structured guidance in scientific work is a basic prerequisite for the successful completion of a doctoral project. The following factors play a crucial role in the successful implementation of the project in the Graduate School: teaching the basic skills of scientific work, providing space for participants to support and exchange ideas with each other, and supporting participants in their personal development so that they feel equipped for a scientific and professional career. All these factors will be taken into account when creating and developing the curriculum.

## Data Availability

The raw data supporting the conclusions of this article will be made available by the authors, without undue reservation.
