# Supplementary material for: Expectations of doctoral students in the field of medicine and health sciences towards a graduate school: an online cross-sectional survey in Germany
Source: Front Med (Lausanne). 2024 Dec 5;11:1481796. doi: 10.3389/fmed.2024.1481796 (PMC11655207; doi:10.3389/fmed.2024.1481796)
Supplement: Supplementary file 1 [file Table_1.docx]

**Supplementary 1**

**Excerpt from the Questionnaire #1: Wishes and expectations of a Graduate School**

Wishes and expectations of a Graduate School (a Graduate school is a thematically focused programme for the supervision of doctoral theses, usually limited in time)

Please select the appropriate answer for each point:

I would expect the following from structured doctoral supervision in a Graduate School…

|  | strongly disagree | disagree | neither agree nor disagree | agree | strongly agree |
| --- | --- | --- | --- | --- | --- |
| to find a (therapeutic) career perspective for myself |  |  |  |  |  |
| to find an entry into a scientific career for myself |  |  |  |  |  |
| individual guidance for scientific work |  |  |  |  |  |
| support in literature research |  |  |  |  |  |
| guidance in managing  literature (selection, literature management) |  |  |  |  |  |
| introduction to research methodology and study design (e.g. clinical trials, meta-analysis, comparative effectiveness research, qualitative research, basic research, evaluation of complex interventions, whole systems analysis) |  |  |  |  |  |
| software training (e.g. Word, Excel, SPSS) |  |  |  |  |  |
| communication training |  |  |  |  |  |
| conflict management |  |  |  |  |  |
| promotion of personal development in science |  |  |  |  |  |
| in-depth familiarisation with the various therapeutic approaches and their principles |  |  |  |  |  |
| networking with other doctoral students |  |  |  |  |  |
| mutual support in case of questions or problems |  |  |  |  |  |
| opportunity for interdisciplinary exchange within the Graduate School |  |  |  |  |  |
| learn about epistemological and health-theoretical approaches |  |  |  |  |  |
| motivation for the writing process and persevere during the doctoral phase |  |  |  |  |  |
| individual supervision by a statistician to better understand the data |  |  |  |  |  |
| quality improvement of the doctoral theses |  |  |  |  |  |
| better final grade |  |  |  |  |  |
| receiving a predicate due to participation |  |  |  |  |  |
| assumption of all costs associated with Graduate School programme |  |  |  |  |  |
| assumption of material costs (for the doctoral thesis) |  |  |  |  |  |
| assumption of travel costs (for the doctoral thesis) |  |  |  |  |  |
| scholarship program of its own (monthly maintenance allowance) |  |  |  |  |  |

Please select all applicable answers:

The programme or supervision in the Graduate School should ...

- take place at different locations
- provide personal counselling and support
- take place predominantly in group work

contain modules for personal development (e.g. stress management, biography work)

- other:

Video conference: How often would you attend an event per year?

In attendance: How often would you attend an event per year?

At different university locations: How often would you attend an event per year?
