# Supplementary material for: Expectations of doctoral students in the field of medicine and health sciences towards a graduate school: an online cross-sectional survey in Germany
Source: Front Med (Lausanne). 2024 Dec 5;11:1481796. doi: 10.3389/fmed.2024.1481796 (PMC11655207; doi:10.3389/fmed.2024.1481796)
Supplement: Supplementary file 2 [file Table_2.docx]

Tab. 4 A sample of free text answers documented additional items to predefined item list, providing new aspects.

| \| In addition, I would expect the following support from a graduate school…….. \| \| --- \|   “Coordination of offers of doctoral theses, moderation of discussions between doctoral student and doctoral supervisor, if necessary.” |
| --- | --- |
| “… either participation is free of charge, but then I also do not expect any coverage of material or travel costs as requested above. Or if there are costs, then I also expect that something is provided for this financial investment (high-quality programme, support in financing, e.g. …...”. |
| “A professionally solid backing without hindering the own scientific development of the doctoral student (e.g. making contacts independently. Personality development, motivation, etc. then develops by itself in my view and should not be part of a graduate school.” |
| “Perspectives in other areas -> Looking beyond the box Retreat/symposium for mutual presentation of the work, so that presentations for congresses or similar are already practiced and a reduction to the most important, as well as a structure for the monograph.” |
| “Access to experts for many different topics” |
| “To have a permanent contact person outside of one's own work group in order to be able to discuss possible problems impartially.” |
| “Topical and methodical lectures. Opportunities for peer-to-peer lectures.” |
| “Learning presentation techniques - Speaker course - mediating contacts with research institutions” |
| “to have the possibility to get in contact outside the meetings in case of questions, exchange of experiences by alumni” |
| “Since I was working in other jobs and also had a family with two children to take care of, it was good for me that I did not have to participate in a graduate programme. Also in view of the many strokes of fate in my family, it was good for me that I was free to organize my own life and not have to meet obligatory deadlines that I would certainly not have been able to keep.” |
| “Support in the acquisition of funding and applications (e.g. ethics committee)” |
